# Enhancing Blood Cell Diagnosis Using Hybrid Residual and Dual Block Transformer Network

**DOI:** 10.3390/bioengineering12020098

**Published:** 2025-01-22

**Authors:** Vishesh Tanwar, Bhisham Sharma, Dhirendra Prasad Yadav, Ashutosh Dhar Dwivedi

**Affiliations:** 1 Chitkara University Institute of Engineering and Technology, Chitkara University, Rajpura 140401, Punjab, India; vishesh.tanwar@chitkara.edu.in; 2 Centre of Research Impact and Outcome, Chitkara University, Rajpura 140401, Punjab, India; 3 Department of Computer Engineering & Applications, G.L.A. University, Mathura 281406, Uttar Pradesh, India; dhirendra.yadav@gla.ac.in; 4 Cybersecurity Group, Department of Electronic Systems, Aalborg University, 2450 Copenhagen, Denmark; addw@es.aau.dk

**Keywords:** leukemia, classification, residual network, dual attention, vision transformer

## Abstract

Leukemia is a life-threatening blood cancer that affects a large cross-section of the population, which underscores the great need for timely, accurate, and efficient diagnostic solutions. Traditional methods are time-consuming, subject to human vulnerability, and do not always grasp the subtle morphological differences that form the basic discriminatory features among different leukemia subtypes. The proposed residual vision transformer (ResViT) model breaks these limitations by combining the advantages of ResNet-50 for high dimensional feature extraction and a vision transformer for global attention to the spatial features. ResViT can extract low-level features like texture and edges as well as high-level features like patterns and shapes from the leukemia cell images. Furthermore, we designed a dual-stream ViT with a convolution stream for local details and a transformer stream for capturing the global dependencies, which enables ResViT to pay attention to multiple image regions simultaneously. The evaluation results of the proposed model on the two datasets were more than 99%, which makes it an excellent candidate for clinical diagnostics.

## 1. Introduction

In the United States of America, leukemia is one of the most significant contributors to mortality among all cancers, as it impacts the blood and bone marrow. Annual deaths from leukemia exceed 300,000 worldwide, and over 450,000 newly diagnosed cases are reported each year, according to recent reports on global health. There is no doubt that the disease significantly affects lives, not only due to the deaths caused, but also by considering how incredibly heavy the burden is for healthcare systems and families. Even with this information, early and accurate detection continues to be a significant challenge that highlights how much better our diagnostic tools must become [1].

Leukemia is characterized by the proliferation of abnormal white blood cells with decreased normal blood cell production. There are four types of leukemia: acute lymphoblastic leukemia (ALL), acute myeloid leukemia (AML), chronic lymphocytic leukemia (CLL), and chronic myelogenous leukemia. Lining the blood vessels are lymphoid cells that help the body produce antibodies and assist in immune support, which is why ALL is a disease that becomes widespread from one point to another point in the limbs. The cause is not well-defined, but mutations are generally inherited with syndromes and environmental factors. Manifestations may include fatigue or tiredness, recurrent infections, bruising or bleeding easily, pain in the bones and joints, and an enlargement of lymph nodes. Diagnosis is usually through performing blood tests that indicate a high concentration of immature lymphocytes, bone marrow biopsy, and tests to identify specific mutations [2]. Acute myeloid leukemia is an aggressive form of leukemia with most of its effects on myeloid cells, which mature into different forms of blood including red blood cells, platelets, and some white blood cells [3]. Myeloblasts are cells meant to develop into granulocytes, but in AML, they do not mature well and pile up. However, a history of smoking and genetic disorders such as myelodysplastic syndromes and previous exposure to chemotherapy, radiation, or certain chemicals and their risk factors for developing AML can be noted. The treatment generally involves a course of intensive chemotherapy that aims to induce remission, followed by post-remission therapy in the form of further chemotherapy, targeted therapies, or stem cell transplantation. Recently, the development of compounds based on targeting specific mutations has transformed the outcomes for some subtypes of AML [4].

Leukemia develops over many years, and patients may live without symptoms for much longer before becoming ill. The leukemia cell is diagnosed with blood tests that show increased white cell count, flow cytometry, and sometimes bone marrow biopsy. Treatments are not started until symptoms develop or the disease has advanced. Fortunately, therapies exist to treat advanced or metastatic NSCLC including chemotherapy, immunotherapy, and targeted therapy based on specific molecular abnormalities that drive the growth of these cancers. Stem cell transplants may be an option in select cases [5]. These may be distinguished as acute leukemia of both the lymphoid and myeloid types (ALL, AML), and chronic leukemia is mainly small-lymphocytic cells of either B-cell or T-cell origin (CLL is found in most affected patients with a detectable MAP level). The same principle applies to the early detection of the leukemia matrix for the proper treatment and a greater chance of survival among patients. Conventional means of diagnosing leukemia are based on microscopic inspection and flow cytometry, which takes a lot of time and may lead to human mistakes [6].

Recent studies have shown that using convolutional neural networks (CNNs) and GAN (generative adversarial network) have shown great results in the medical imaging domain [7]. Indeed, CNNs have a bias due to the nature of local feature extraction. On the other hand, the vision transformer (ViT) model also delivers state-of-the-art performance [8]. In this study, we assessed the classification performance in recognizing leukemia cells through ResViT, and hypothesized that modeling with a self-attention mechanism can detect features at both the local and global levels in the cell images, eventually leading to increased accuracy rates of detecting leukemia. The dual-stream block in ViT enables the model to focus on different regions in an image concurrently. Furthermore, we evaluated the model on the two datasets and compared the results with other methods.

The contribution of the proposed ResViT method is as follows.

(1) We integrated ResNet-50 and ViT to diagnose leukemia cells efficiently. The ResNet-50 extracts local high-dimension spatial features, and ViT provides global attention to the spatial features.
(2) We implemented convolution and the ViT stream for local and global attention. The CNN block extracts local spatial attention and the ViT block provides global attention to the different regions of the leukemia cells.
(3) The performance of the ResViT was tested on two open-source datasets and compared with several other methods.

The rest of this paper is organized as follows. Section 2 presents the related work in leukemia cell classification. The architecture of the ResViT is elaborated in Section 3. We highlight our experimental results in Section 4, and Section 5 presents the findings and their implications, followed by our concluding remarks on future research in Section 6.

## 2. Related Work

Amin et al. [9] applied a ViT for global attention on spatial features using self-attention between image patches to process visual patterns. Their model achieved an accuracy of 81.5%. Chen et al. [10] introduced a novel automated approach for blood cell classification via the shifting window vision transformer. The window-based transformer mechanisms enhanced the categorization results and their model attained an accuracy of 87.29%. However, fine-tuning the entire model resulted in a substantial difference above 10% in overall performance. Perumal and Murukessan et al. [11] developed a new approach for classifying malignant cells from MRI. The procedure is not easy, since the formation of both cells is quite similar. Flow cytometry is an advanced technology with a hefty price tag, which is in demand for early diagnosis but is impossible in all places. Automated diagnostic tools for diagnosis can improve the low-cost microscopic image data. Therefore, an attention-based neural network as the residual transformer architecture of that classification was proposed, and the model outperformed the benchmark ISBI 2019 challenge dataset, achieving an F1-score of 84.89%.

Katar et al. [12] created an interpretable ViT model to detect WBCs in blood film, which utilized a self-attention technique to extract information from the input images. Using the 16,633 samples for training and validation, they could categorize WBCs with a 99.40% accuracy rate. Ali et al. [13] investigated the performance of ViT and CNN through transfer learning in an auto bootstrapper for PBS reading. The examination of PBS, PBC, and BCCD datasets was converted into comparable datasets to evaluate the impact of data volume and noise resilience on the corresponding neural networks. Findings on PBC indicate that ViT is an optimally situated deep learning solution in scenarios of data shortage. The BCCD results demonstrate that ViT outperformed the CNNs of ImageNet due to its superior ability to handle filthy, noisy data on the image. This was due to its ability to learn both global and local features as well as utilize the residual connection with additional time and computational complexity. In their paper, Wang et al. [14] applied convolutions with token embedding to substitute for positional encoding that had coarse spatial information. After ViT, a sparse attention module for localizing parts within the image was used to promote the model further and describe the fine-grained features. Finally, one study enhanced the intra-class consistency and inter-class difference of characteristics regarding classification by a contrastive loss function. Sunita et al. [15] proposed a model for leukemia detection using the ResNet-50 model and achieved 100% accuracy on the ALL1IDBI dataset.

Gokhale et al. [16] first converted image representation through a stacked autoencoder followed by the improved deep insight algorithm. Then, the translated data were fed into the vision transformer to construct the classification model. A framework for leukemia classification named CoTCoNet with the dual-feature extraction pattern was proposed by Raghaw et al. [17], incorporating both the global and fine-grained patterns. This included a graph-based module for the hidden features’ discovery of leukocyte cells. The results showed that it significantly outperformed any approach in prior works with an accuracy of 98% and F1-score of 98.93% on a dataset of 16,982 annotated cells. Tarimo et al. [18] introduced a novel technique that could be applied in identifying and classifying different types of white blood cells. The two-way technique employed two varieties of images, WBCs and the nucleus, and was successfully integrated across 16 classes in the YOLO and ViT. Their model showed an excellent classification accuracy rate of 96.449%. Chen et al. [19] presented a solution to boost leukocyte classification. Their model showed very nice generalization over other datasets; it is more robust toward optimizers and focused well on discriminative parts of different cells. Leng et al. [20] developed a pure transformer-based end-to-end object detection network for leukocyte detection. Such an advanced model was then trained on challenging common objects in context to gain pre-trained weights. The advanced DETR could rise to a mean average precision detection performance of 0.961 and better than that of CNN. Leng et al. [21] designed an AMLNet lightweight classification model for acute myeloid leukemia, where their model improved the performance of the CNN and transformer. The data augmentation approach and model structure helped improve the classification performance. AMLNet achieved 0.9935%, 63.91%, and 96.43% in AUC, sensitivity, and accuracy, respectively, and outperformed other methods on other public datasets. Guetarni et al. [22] introduced an approach for bone marrow cells that employed integrating multi-scale information. The technique yielded an accuracy of 94.41%, which surpassed that of existing approaches. Ali et al. [23] combined cross-former and class centroid learning to acquire multi-scale information from cell images, enhancing the distinction of feature recognition ability in images and rejecting low-confidence predictions. Their method achieved a recognition accuracy of 94.41%. Meanwhile, the CCL was the trusted cooperative algorithm that successfully rejected 21.07% of cell images, corresponding to poor prediction confidence results.

Traditional methods used for leukemia diagnosis are not precise in identifying the cell morphology, especially when it comes to subtyping. Many of the existing methods for leukemia cell classification suffer from several limitations. For instance, models are often poor in challenging cases where leukemia subtypes have subtle morphological differences. For example, Amin et al. [8] only obtained an accuracy of 81.5% with a basic ViT model, and Chen et al. [9] improved it to 87.29% using a shifted window transformer. Computational inefficiencies also enter into the picture because classical vision transformers and models require high resources and large fine-tuning periods, making them impractical for real-time clinical environments. Generalization also posed a challenge because the CNNS used by Ali et al. [12] showed a degraded performance when exposed to noisy or limited datasets, while standalone ViTs tended to overfit. Here, CNNs performed well in local feature extractions but failed to acquire global spatial understanding, while ViTs may not have captured the fine-grained patterns. Furthermore, issues of class imbalance and misclassification have not been ruled out. For instance, window-based transformers suffer against imbalanced datasets or morphologically similar cell types.

We utilized ResNet-50 for the high dimensional spatial features of leukemia cells. Afterward, the extracted features were flattened, and tokens were generated for input to the dual stream. For local and global attention to the feature map, we divided the query (Q) into two halves and passed them to the convolution and transformer stream. In this way, our model reduced the computation costs of the attention mechanism and focused on the edge and boundary region of the leukemia cells.

## 3. Proposed Method

**Figure 1:**
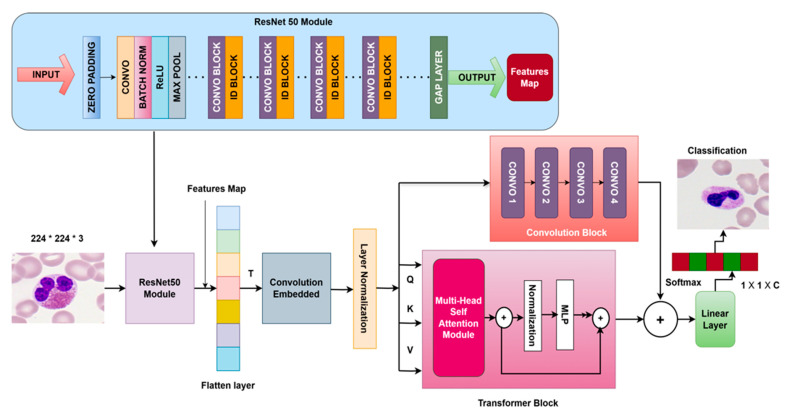
Proposed ResViT architecture for the diagnosis of leukemia cells.

ResNet-50 extracts low-level information such as edges and textures and high-level patterns relevant to cell-type distinction. This feature extraction results in a rich feature map combining all of the details from various abstraction levels. After extracting hierarchical features from ResNet-50, they are fused into a rich feature map, combining low-level, mid-level, and high-level abstractions. This fused feature map is tokenized and serves as an input to the dual-stream vision transformer. Within the dual-stream architecture, two streams are established: a convolutional stream for the tokenized feature map, and a transformer stream for the transformed tokens. We processed tokens through the convolutional layers, capturing more local details, fine textures, and cell boundaries. Multi-head self-attention was applied to the transformer stream, which captured the spatial and long-range dependencies involving the cell image and spatial relationships among the different parts, giving a sense of global context associated with several parts of the images. ResViT thus enabled us to look at the cell structure of the micro-level details and discriminate complex changes between these leukemia cell types. Figure 1 shows the proposed ResViT architecture for the diagnosis of leukemia cells.

### 3.1. ResNet50 for Spatial Feature

ResNet-50 is a deep convolutional neural network architecture with 50 layers that was designed to overcome the long-standing vanishing gradient problem in very deep neural networks. The concept of residual learning was discovered with the help of skip connections, where the network bypasses one or more layers. These skip connections ease the training of deeper networks, allowing gradients to pass back through previous layers in an effective manner and work against degradation problems due to the depth caused by increased networks. ResNet50 is built from stacks of residual blocks, comprising convolutional layers topped with a batch normalization function along with an activation function, hence powerful enough to extensively extract complex feature patterns from the input images. The architecture uses 1 × 1, 3 × 3, and 1 × 1 convolutional filters so that the reduction in computationally expensive calculations does not sacrifice the major features. Due to its outstanding performance on various benchmarks in computer vision, high accuracy, and efficient training, ResNet50 has become a highly widespread model for a wide range of image recognition tasks.

Let input X pass to the convolutional layer, which extracts the spatial described in Equation (1):

X_1_ = ReLU (Conv2d (X, W_1_) + b_1_) (1)

where X_1_ is the output feature map from the convolution layer, and W_1_ and b_1_ are the weight and biases of the convo layer, respectively. ReLU is the activation function applied element-wise to introduce nonlinearity.

As the image travels through the multiple residual blocks of ResNet-50, each block extracts increasingly abstract and complex features. These layers focus on capturing the various cellular and sub-cellular structures by learning the hierarchical representations, which are essential for identifying the nuanced morphological characteristics of leukemia in blood cells. The residual connections within ResNet-50 preserve crucial details that could be lost in deeper layers, making it effective for capturing both low-level and high-level features.

In each residual block, the input is added to the output of convolutional layers to create a “residual” connection mathematically, as shown in Equation (2):

X_i+1_ = F (X_i_, W_j_) + X_i_ (2)

where F (X_i_, W_i_) is a series of convolutions within the block, as shown in Equation (3). This helps capture the various levels of features like low-level features (Flow), mid-level features (F_mid_), and high-level features (F_high_):

F (X_i_, W_i_) = ReLU (Conv2d (X_i_, W_i_) + b (3)

Each block’s X_l_ output becomes the next block’s input, as shown in Equation (2), progressively refining features. This process continues through the network’s layers, capturing different levels of abstraction from the textures and shapes to complex cellular structures, and the final feature map is obtained to concatenate all levels of features into a single feature map, as shown in Equation (4).

X_final_ = F_low_ ⨁ F_mid_ ⨁ F_high_ (4)

The final residual block, the global average pooling layer, condenses the feature maps (X_final_).

### 3.2. Token Generation

The extracted feature is flattened and tokenized for input into a dual-stream vision transformer. In this architecture, tokens are split into two streams to capture the local and global contexts. The convolutional stream processes tokens with convolutional layers, focusing on local details such as textures and shapes. Meanwhile, the transformer stream uses multi-head self-attention to relate different parts of the image, capturing global spatial relationships. This allows the model to effectively understand both minute patterns and large-scale contextual information. We generated tokens using conv-embedding, as shown in Equation (5):

T = ReLU (Conv2d (X_final_, W_1_) + b_1_ (5)

where W_l_ and b_l_ are the weights and biases for the convolutional layer used to generate tokens, respectively. Each token now represents a part of the X_final_ feature map.

### 3.3. Dual-Stream Vision Transformer

The vision transformer was initially created for NLP (natural language processing). It possesses significant potential for gaining both global and local attention, which can be leveraged for implementing various disease diagnoses, and has recently been utilized in remote sensing, precise agriculture, and disease diagnosis. In the proposed study, we utilized ResNet-50 for high-dimensional spatial features and a dual-scale ViT encoder for local and global attention to the feature map. In the dual scale, one scale contained a depth-wise convolution layer for local attention, and the other had a ViT encoder for global attention. Initially, feature pools derived from the ResNet50 model were transmitted to the 2D convolution block for creating tokens using Equation (5). From the token query (Q), the key (V) and value (V) can be generated, as shown in Equation (6).

Q = TW_q_, K = TW _k_, V = TW_v_ (6)

where W_Q_, W_K_, and W_V_ are the learned projection matrices. Furthermore, the query Q is portioned into two parts, $q_{1}\in R^{M\times C/2}$ and $q_{2}\in R^{M\times C/2}$. After that, q_2_ and q_1_ are passed to the transformer and convolution block, respectively. By doing this, the computational resource utilization is reduced significantly. The q_1_ tokens are passed through lightweight convolutional layers to capture local information, as mathematically defined in Equation (7):

Conv(q1) = BatchNorm (Conv2d (q_1_, w_c_, b_c_) (7)

where w_c_ and b_c_ are the weights and biases of the convolution layer, respectively. A global mechanism was incorporated into the attention block to capture the image’s global contextual details. The output generated from this block was adjusted to align with the dimensions of the convolution block, and attention was computed as follows.

Attn(Q_2_) = Reshape (Softmax (Q_2_K_t_/√d_k_) V) (8)

where d_k_ is the dimension of the key, and the softmax operation helps compute the attention scores for global information extraction. Finally, we combined the local and global attention obtained from the convolution and transformer block as follows:

F_final_ = Concat (Conv(q1), Attn(q2)) (9)

The F_final_ tensor passes to the classification module. The softmax layer predicts the leukemia cell by the class labeling variable j, weight W_fc_, and bias b_fc_ available in each iteration, and is defined as follows:

(10) $$\hat{y}=SOFTMAX\;(W_{fc}F_{final}+b_{fc})$$

We calculated the loss of the model on each dataset using the categorical-cross-entropy function.

## 4. Results

In this section, we discuss the experimental results of the ResViT proposed model on two datasets. The ResViT model addresses the challenge of distinguishing subtle morphological differences that may make it difficult to identify subtypes of leukemia. Most traditional methods and models often fail in this regard, since most leukemia subtypes appear to be very similar under a microscope. ResViT resolves this by taking a two-tiered approach: it can not only take on fine details like the textures of cells and cell boundaries with convolutional layers, but understand the bigger picture, the spatial relationships, through self-attention in the vision transformer. This local and global analysis would help the model catch the most minute features distinguishing one subtype from another.

### 4.1. Datasets

This study used the two-datasets first, consisting of 18,236 single-cell images from peripheral blood smears of 200 subjects captured with an M8 digital microscope/scanner (Precipoint GmbH, Freising/Germany) at 100× magnification and a resolution of 14.14 pixels per micron. These pictures were organized into 15 different morphological cell types of leukemia: basophil (BAS), erythroblast (EBO), eosinophil (EOS), keratinocyte stem cell (KSC), lymphoblast-A (LYA), lymphocyte (LYT), myeloma cell Z (MMZ), monoblast (MOB), monocyte (MON), myeloblast (MYB), myeloid cell (MYO), neutrophil band (NGB), neutrophil segmenter (NGS), promyeloblast (PMB), and promonocyte (PMO) [24]. This DS2 ALL dataset comprises 20,000 images, and they can be divided equally into four classes. In each class, the number of images was 5000 to balance the dataset. These class labels with their descriptions are shown as follows. (1) ALL_B: This class includes images that are considered to be peripheral blood smears with a benign label, which means no signs of leukemia. (2) ALL_E: This was class selected as representative for the very early stages of ALL. (3) ALL_Pre: This encompasses the images of a peripheral blood smear of patients before ALL, thus underlining a leukemia condition. (4) ALL_Pro: This category encompasses images from the pro-ALL stage, which is at an even more advanced state of ALL [25]. Table 1 presents sample pictures with their corresponding name labels for every type of leukemia cell.

### 4.2. Experimental Settings

The script for the suggested method was developed in Python 3.9, utilizing TensorFlow 2.0, and executed on a Windows 11 system equipped with an Nvidia GeForce GTX TITAN X GPU and 128 GB of RAM. Nvidia designs and manufactures the GeForce GTX TITAN X GPU as part of its GeForce GTX series and company is based in Santa Clara, California. The batch size, epochs, and learning rate were set as 32, 140, and 0.0001, respectively. Given the asymmetry in the dataset, we used fivefold cross-validation to prevent skewed performance assessments. In each fold of cross-validation, 80% of the pictures were utilized for training, while the remaining 20% were used for validation.

### 4.3. Quantitative Results

**Figure 2:**
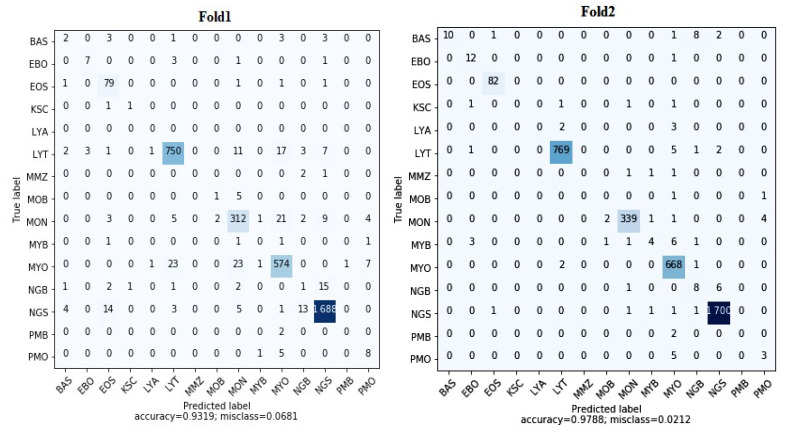

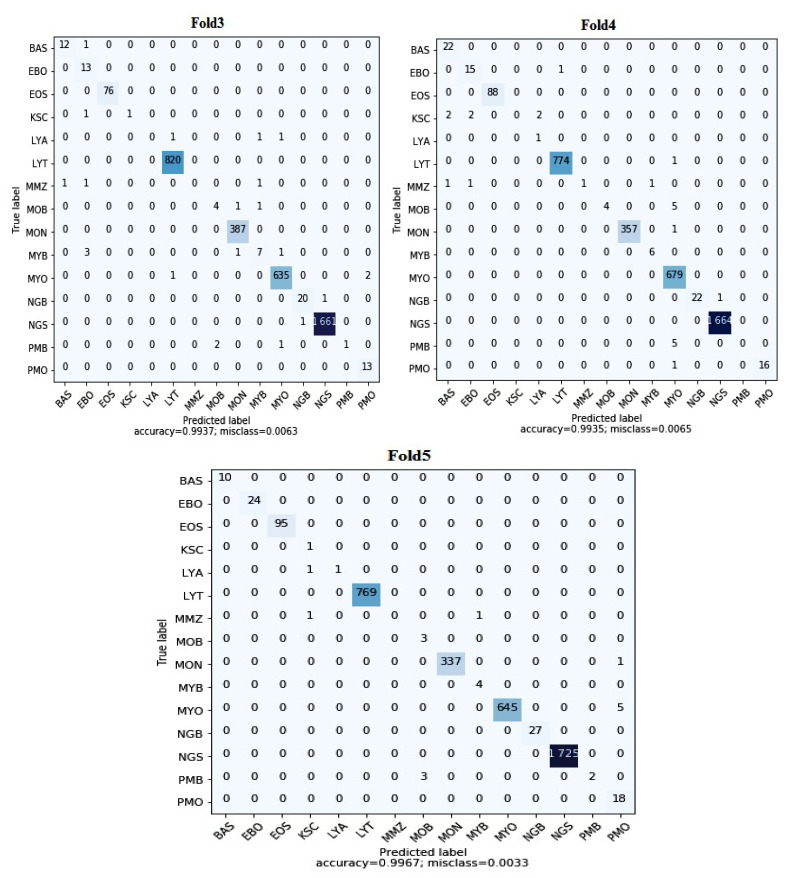
Confusion matrices obtained from each of the 5-fold cross-validations on dataset-1.

We evaluated ResViT on dataset-1 using 5-fold cross-validation, and the result of each fold is presented in Figure 2. We noted that in fold1, the model had 104 FP (false positive) and 140 FN (false negative) values whereas in fold2, the model had 44 FP and 23 FN values. Furthermore, in fold3, the FP and FN values decreased to 11, whereas in fold5, the model had 7 FP and 4 FN values. Table 2 gives the results of the proposed ViT model for classifying leukemia cell types by 5-fold cross-validation. With average precision and recall at 64.73% and 67.11%, respectively, and F1-score at 65.84%, the model was seen to be quite capable, but has vast potential for improvement. Kappa values ranged from 0.902 to 0.995, and overall, the agreement was extremely strong beyond chance, though in fold5, fold4, and fold3. Misclassification rates were generally low. The overall average was 0.0086, though fold1 showed a surprisingly higher rate of 0.0681. Between fold5, fold4, and fold3, the accuracy was the highest at over 99%. Accuracy decreased in fold2 and fold1 to 97.87% and 93.19%, respectively, and it may well be that there were some splits or types of cells that really posed a serious challenge for the model. The average accuracy for the ViT model was 97.88%.

**Figure 3:**
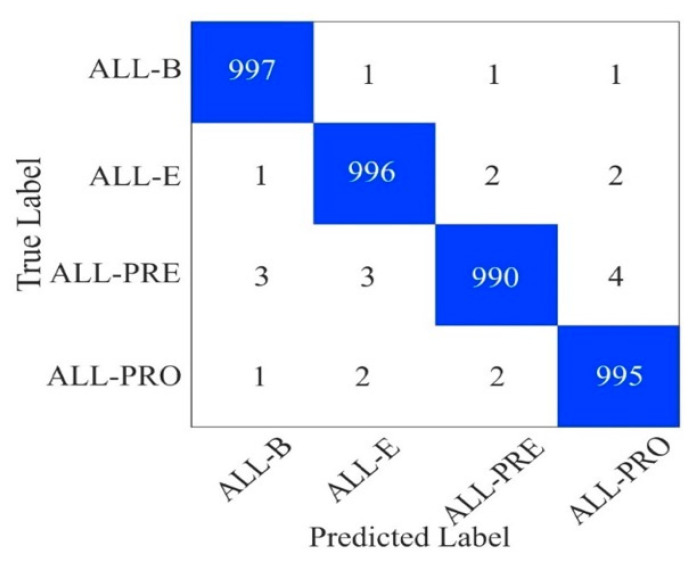
Confusion matrices of dataset-2.

Furthermore, we applied a data augmentation technique to increase the size of dataset-2. After, augmentation, each class contained 5000 images. ResViT was trained for 140 epochs, and the confusion matrix was plotted for the test dataset. Figure 3 shows the confusion matrix of ResViT on dataset-2. True labels on the Y-axis are the actual leukemia categories, and the predicted labels on the X-axis relate to the model’s classification. High diagonal values (997, 996, 990, and 995) represent correct predictions of the majority of the cases for each subtype. Off-diagonal values signify misclassifications that were very small in number. For example, in the ALL-B row, it indicate one misclassification for ALL-E, ALL-PRE, and ALL-PRO, while for ALL-PRO, the classifier mislabeled one case as ALL-B, and two as ALL-E. Generally, the ViT model correctly classified 997 cases of ALL-B out of 1000, ALL-E out of 1000, ALL-PRE 990 out of 1000, and ALL-PRO 995 out of 1000. Therefore, it is clear that the ResViT model can distinguish these types of leukemia with very few errors at a highly accurate level.

Table 3 presents the performance metrics of ResViT on dataset-2 for classifying acute lymphoblastic leukemia (ALL) into four subtypes: ALL-B, ALL-E, ALL-PRE, and ALL-PRO, with key metrics including the recall, precision, F1-score, Kappa1, and accuracy. The model demonstrated high recall (e.g., 99.50% for ALL-B), meaning that it accurately identifies most of the true cases, and high precision (e.g., 99.70% for ALL-B), indicating that most of the predicted cases were correct. The F1-scores, which balance recall and precision, were also high across all subtypes (e.g., 99.50% for ALL-B and 99.24% for ALL-PRE), reflecting strong overall classification performance. Kappa values, such as 99.92% for ALL-B, showed near-perfect agreement between the predictions and actual labels, indicating that the model performed significantly better than chance. The accuracy for ALL-B was 99.42%, further demonstrating the model’s reliability. Overall, the ViT model showed exceptional performance with minimal misclassifications, making it highly effective in distinguishing between ALL subtypes.

## 5. Discussion

In this section, we present the discussion of the model performance and other statistical parameters for model generalization. ResViT benefits from the ResNet50 capability of high-dimension spatial features as well as the dual-stream ViT encoder, which provides local and global correlation on the spatial feature map. We evaluated our model performance on the two datasets and obtained notable accuracy. The high values of the AUC scores and accuracy for basophils and myeloblasts showed that both cell types had a characteristic morphology with particular morphological features easily identified by the attention mechanisms of the model. On the other hand, for classes such as neutrophil bands, it had slightly less AUC and accuracy values among the cell types that were likely to be morphologically less differentiated. Table 4 shows a summary of the deep CNN and ResViT on different datasets.

### 5.1. Accuracy and Loss Graph

**Figure 4:**
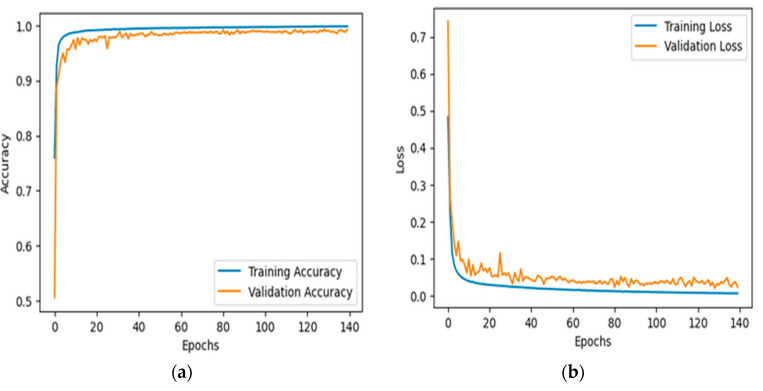
The accuracy and loss over the training and validation datasets1 are presented in (**a**) and (**b**), respectively.

Figure 4a depicts the accuracy of the proposed model, trained and validated on a ResViT model in terms of the classification of leukemia over 140 epochs on datset1. The blue curve in this figure refers to the training accuracy of the model; from the plot, it can be seen that the rises in accuracy for this model were steep, peaking close to values of 100%. This means that the model learned well enough. The orange curve is the performance of the model on validation and reflected a very nice growth at early epochs that did not reach the same level as that during training. It oscillated slightly after about 20 epochs, showing variability in how well the model generalized into unseen validation data. Thus, the results prove that the model was very accurate at approximating the training data, however, there was a possibility of overfitting, since the fluctuation of the validation accuracy was quite rampant and sometimes performed worse on the validation dataset, but the performance could be kept at high levels overall. It also presents evidence of how the proposed ResViT model is robust enough to exploit for leukemia classification.

Figure 4b illustrates the loss of the proposed model on the task of leukemia classification over 140 epochs using the ResViT model on dataset-1. The blue curve, training loss, started high at the start of training but dropped quickly through the first few epochs. It looked like it was really effective at learning because it was still learning into later epochs. At that point, the training loss leveled off to an almost immeasurably small value, suggesting a negligible error against the training dataset. The orange curve represents the validation loss. Similarly, the validation loss showed a sharp fall at first, but was still much higher than the training loss. From there, after the sharp drop at the beginning, it oscillated slightly around some very low values, meaning a good generalization performance. While the training loss was persistently and constantly plummeting and stabilizing, there were some fluctuations in the validation loss, which may suggest slight generalization issues with the model, although it generalized quite well on both the training and validation sets.

**Figure 5:**
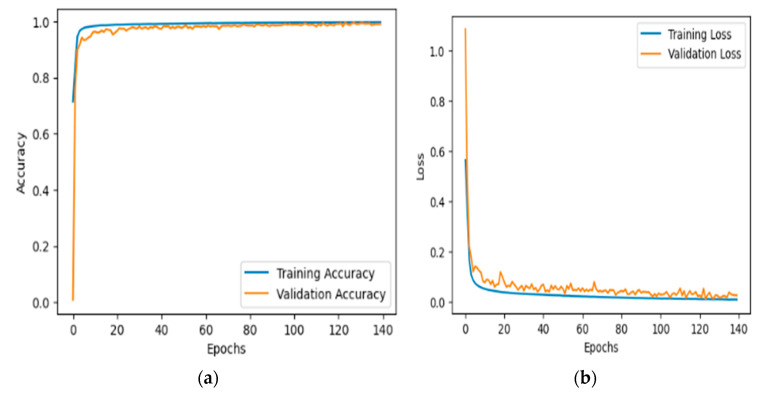
The accuracy and loss over the training and validation dataset-2 is presented in (**a**) and (**b**), respectively on.

Figure 5a,b presents two plots showing the training and validation accuracy (a) and the training and validation loss (b) for a model trained on the dataset-2 for acute lymphoblastic leukemia (ALL). In the accuracy plot, both the training (blue) and validation (orange) curves rapidly increased during the first 20 epochs and stabilized near 1.0, indicating that the model achieved near-perfect accuracy for both datasets. The close alignment of these curves suggests strong generalization without overfitting. In the loss plot, the training loss initially decreased sharply, while the validation loss stabilized and fluctuated slightly. The small gap between the curves further indicates that the model generalized well to the validation set, with minimal overfitting. Overall, the plots demonstrate that the model performed effectively, achieving high accuracy and stable loss on both the training and validation datasets.

### 5.2. Receiver Operating Characteristics of Cell (ROC) Curve

**Figure 6:**
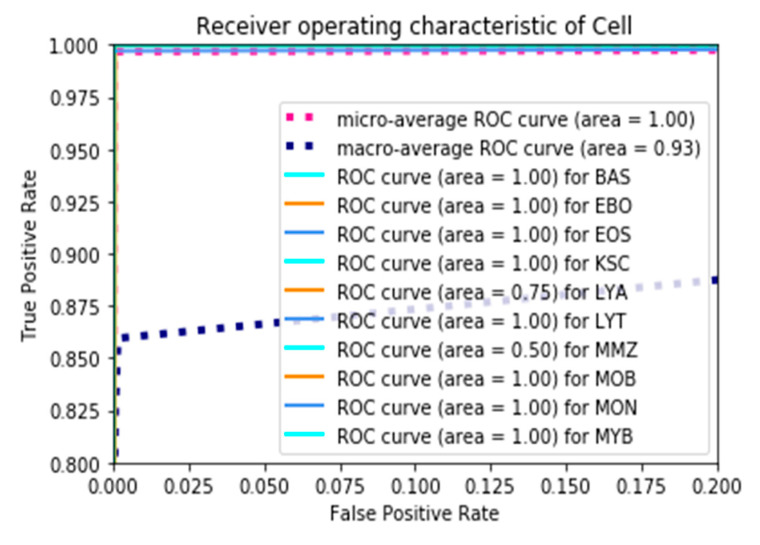
ROC curves for the proposed vision transformer (ViT) model in the classification of 15 leukemia cell types.

Figure 6 presents the assessment of the ROC curves concerning the proposed ResViT model for the classification of 15 different types of leukemia cells. The micro-average ROC curve had an AUC of 1.00, which means that the model totally worked in classifying all of the classes combined by true positives from all classes with false positives. The AUC of the macro-average ROC curve was 0.93, speaking to the strong overall performance regardless of the class by treating the classes as equal, but showed that the model was lacking by a slight margin for several classes of individuals. The class-specific ROC curves give evidence of perfect discrimination with an AUC = 1.00 for BAS, EBO, EOS, KSC, MON, and MYB, confirming that ViT has excellent ability in classifying these leukemia subtypes.

The AUC dropped significantly to 0.75 for the LYA class, suggesting that the model was not good at distinguishing this class from any other. MMZ and MOB were the most difficult classes for the model, with an AUC of 0.50, suggesting a performance not far from random guessing for these cell types. The proposed ViT model performed very well for most classes of leukemia, with nearly perfect AUC values for many classes. There was some difficulty for a few cell types, namely MMZ and MOB, which might require some more refinements or additional data to be able to better distinguish the challenging categories. Indeed, a high value of micro-average AUC as well as macro-average AUC confirmed the generalization of the model across the vast majority of classes, pointing to a strong potential for practical application in leukemia classification.

### 5.3. Comparison with State-of-the-Art Methods

Loey et al. [26] utilized AlexNet architecture on a dataset of 2820 images and achieved an accuracy of 99.9%. Pałczyński et al. [27] used MobileNet v2 on a dataset of 260 images and reported an accuracy of 97.4%. Maaliw et al. [28] employed InceptionResNetV2, which succeeded with 99.60% accuracy in classifying normal vs. leukemia, and 94.67% in the classification from normal to L3. Das et al. [29] introduced the orthogonal softmax layer with ResNet18, where 368 images achieved 99% accuracy. Wibowo et al. [30] applied EfficientNetV2M with Bayesian optimization, which achieved 91.37% on a larger dataset of 15,175 images, while Lee et al. [31] utilized data from 241 patients with ResNet-50 at a 91.1% accuracy. Verma et al. [31] presented a CNN-SVM hybrid model that achieved an accuracy of 99% using a private database. The deep convolutional neural network, Inception-V3+FC architecture, was used by Das et al. [32] on 15,135 images with an accuracy of 97.34%. Leng et al. [33] applied improved DETR on 10,323 images and obtained 96.10% precision. The proposed ResViT model obtained a state-of-the-art accuracy of 99.67% in a huge dataset of 18,236 images, proving and extending the ability to capture complex patterns within leukemia cell images, making it a robust solution in medical image analysis. Dipto et al. [34] evaluates two variants of Vision Transformer on a pre-augmented dataset of 12,500 images, achieving 83–85% accuracy.

Table 5 compares the performance of the proposed ResViT model to the existing method of Matek et al. [35] in the task of leukemia cell classification. Each row is dedicated to one type of leukemia cell; metrics include precision, recall, F1-score, and the number of images used for evaluation. The ResViT model consistently reached high precision and recall values across most cell classes, indicating its ability to recover leukemia cells with low false positives and false negatives. For instance, for many classes, ResViT attained a precision value of “1.0” with a recall value of “1.0”, thus making fewer classification errors compared with that of Matek et al., which attained variable performance. High F1-scores also suggest the existence of a good balance between the precision and recall values of ResViT, meaning that the model is reliable in classifying leukemia cells for all classes. However, the results obtained in Matek et al.’s model for some classes, like MYO and NGB, were affected by low precision and recall values, as the classification was less accurate in terms of precision and recall. Altogether, better precision, recall, and even F1-score performance of the ResViT model indicate that the model could potentially replace the other existing models in providing more accurate approaches for the diagnosis of leukemia, where accuracy in cell classification by pathologists is somewhat crucial.

**Figure 7:**
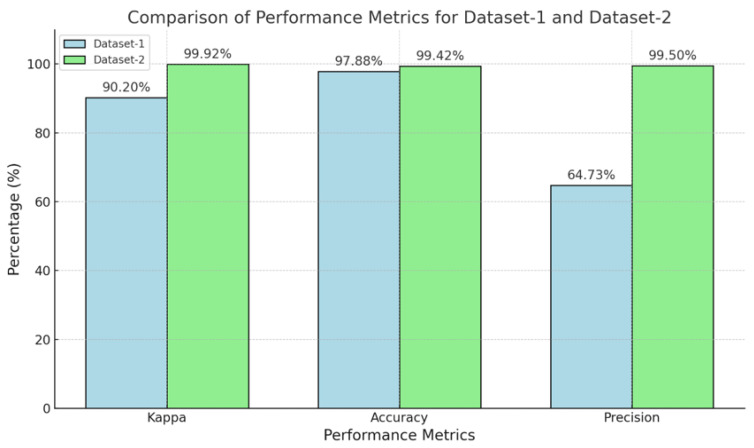
Comparison of the results on two different datasets of the proposed ResViT model.

Figure 7 compares the results obtained from two datasets (Dataset-1 and Dataset-2) using the proposed vision transformer (ViT) model, focusing on three key performance metrics: Kappa, accuracy, and precision. The Kappa score, which measures the agreement between the predicted and actual labels while adjusting for random chance, was 90.20% for Dataset-1, and significantly improved to 99.92% for Dataset-2, indicating better alignment with true labels in the latter. Accuracy, representing the overall proportion of correctly classified instances, was 97.88% for Dataset-1 and rose to 99.42% for Dataset-2, suggesting enhanced performance and fewer misclassifications in Dataset-2.

The precision was notably lower at 64.73% for Dataset-1 but increased dramatically to 99.50% for Dataset-2, implying that the model’s predictions were much more accurate and resulted in fewer false positives for the second dataset. Overall, Figure 7 demonstrates that the ResViT model outperformed Dataset-2 compared to Dataset-1, showing significant improvements across all metrics; the higher Kappa, accuracy, and precision values for Dataset-2 indicate better generalization and reliability, suggesting that this dataset is either more representative or that the model was better tuned to handle it, leading to superior classification performance.

### 5.4. Ablation Study

Our ResViT has two major components: ResNet50 and the dual-scale ViT encoder. ResNet-50 extracts high-dimensional spatial features, while at the same time, the ViT encoder provides local and global attention. In the classical transformer, MHSA (multi-head self-attention) is used for global attention using the query (Q) and value (V), which may miss the local information. In addition, computation is quadratic. Moreover, our ViT encoder contains two streams that calculate the local and global attention through CNN and ViT blocks. We divided the query (Q) into two halves (q/2) on which the attention was calculated. Through this mechanism, we reduced the computation time and improved the attention on the spatial features. Furthermore, we added an ablation study and compared the results of the proposed method with the method proposed by Vaswani et al. [35] and Liu et al. [36] under the same experimental settings. The comparison results are presented in Table 6. We noted that the classical transformer achieved a precision and F1-score of 61.19% and 60.74%, respectively. At the same time, the SwinTransformer proposed by Liu et al. [36] obtained 62.72% and 63.11% in precision and F1-score, respectively. Moreover, the proposed ResViT achieved a precision and F1-score of 64.73% and 65.84%, respectively. In addition, the Kappa and accuracy value of the ResViT were better than those of the transformer-based methods.

Furthermore, we compared the ResViT performance on Dataset-2 with Vaswani et al. [36] and Liu et al. [37] under the same experimental settings, the results of which are presented in Table 7. Table 7 shows that the classical transformer-based method proposed by Vaswani et al. [36] achieved precision and Kappa scores of 94.58% and 95.87%, respectively. At the same time, Swin-Transformer, developed by Liu et al. [37], obtained a 97.12% precision value. Moreover, our ResViT achieved precision and kappa scores of 99.41% and 99.92%, respectively.

#### 5.4.1. Effects of Different Components

We added the effect of different components on the model performance of both datasets, which is presented in Table 8. We evaluated the pure transformer on Dataset-1 and Dataset-2 under the same experimental settings, as discussed in Section 4.2, and the results are presented in Table 8. Table 8 shows that ResNet-50 achieved F1-score and Kappa values of 57.34% and 81.20%, respectively. At the same time, the pure transformer obtained a Kappa and precision of 85.47% and 61.08%, respectively, on Dataset-1, while ResNet50+ViT improved the performance and achieved a 62.82% precision value. Moreover, the proposed ResNet-50+dual stream ViT obtained precision and Kappa scores of 64.73% and 90.20%, respectively. In addition, in dataset 2, ResNet-50 achieved an F1 score and Kappa values of 92.18% and 93.07%. At the same time, ViT obtained an F1 score of 94.95%. Meanwhile, ResNet50+ViT improved the performance and achieved a 97.83% precision value. Moreover, the proposed ResNet-50+dual stream ViT obtained precision and Kappa scores of 99.41% and 99.92%, respectively.

#### 5.4.2. Training and Validation Time Analysis

**Figure 8:**
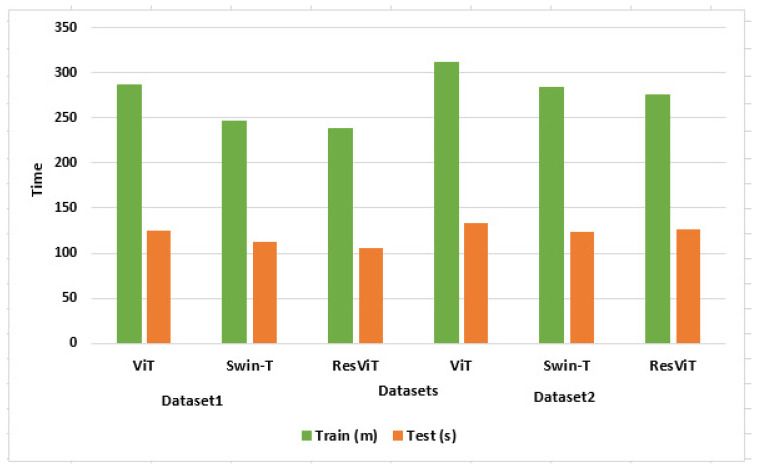
Comparison of the training and validation time on Dataset-1 and Dataset-2.

Diagnosing leukemia traditionally relies on methods like microscopic inspections and flow cytometry, which are not only labor-intensive but also require specialized expertise. Pathologists must closely examine blood smears or bone marrow samples under a microscope, a process that can span several hours or even days, depending on the complexity of the case and the availability of experts. These delays can be critical in clinical settings where time-sensitive decisions are essential for effective treatment. In contrast, the ResViT model streamlines the entire classification process by leveraging the potential of deep learning. After training, ResViT could analyze and classify images within seconds, offering near-instantaneous results. This capability drastically reduces diagnostic delays and ensures a quicker initiation of treatment, which can be lifesaving. ResViT eliminates the need for manual feature extraction, reducing reliance on human intervention. It can classify hundreds or thousands of images simultaneously, accelerating the diagnostic pipeline. We compared the computation time of ResViT with the ViT and Swin-T deveoped by Vaswani et al. [35] and Liu et al. [36] on Dataset-1 and Dataset-2. Figure 8 shows that the classical ViT model proposed by Vaswani et al. [35] took 287 min (m) and 125 s (s) for training and validation, which was the highest in the table due to the computation costs of the MHSA. At the same time, the training and validation time was slightly less in the Swin-Transformer proposed by Liu et al. [36] due to window-based MHSA. Moreover, our ResViT took the least training and validation time due to partitioning the query into two halves and performing attention. In classical ViT, attention is calculated using MHSA (multi-head self-attention), which takes quadratic times. At the same time, the SwinTransformer calculates attention using window-based MHSA. Moreover, our ResViT divides the query into two halves and reduces the complexity. In future study, we will have to design a more lightweight attention mechanism to reduce the computation burden.

## 6. Conclusions

In this study, we designed ResViT for the diagnosis of leukemia cells. Our model extracted high dimensional spatial features from the cells using ResNet50, and attention to the feature map was provided by the dual-stream transformer block. In the dual stream, the query is divided into two parts and fed to the convolution stream and transformer stream for local and global attenti on to the spatial features. Finally, enhanced features are passed to the softmax layer for categorization of the leukemia cell. The experimental results of ResViT on the two datasets were compared with the other ResNet and ViT-based methods. The quantitative results on the two datasets had a performance measure value of more than 99%. The results confirm the practical applicability of the ResViT model in clinical scenarios, making it possible to reach high accuracy and have a small number of misclassifications in different imbalanced datasets. This makes real-time efficient model implementation possible in medical diagnostics, which will improve early leukemia detection, future patient outcomes, and so on. In future studies, we will optimize the model feature map for underrepresented cell types and extend its application to other hematological conditions. In addition, another lightweight attention mechanism in the ViT block can be applied to reduce the computation burden of the system.

## Figures and Tables

**Table 1:** Sample images and labeled name of each leukemia call.

Cell Name (Label)	Sample Image	Cell Name (Label)	Sample Image
Lymphocyte (LYT)	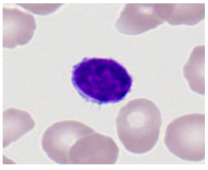	Monocyte (MON)	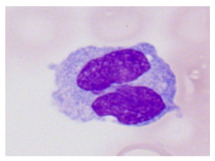
Eosinophil (EOS)	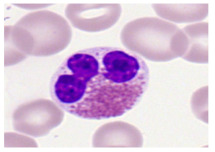	Myeloblast (MYB)	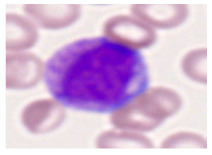
Monoblast (MOB)	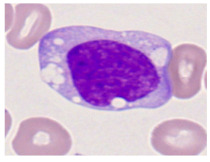	Myeloid cell (MYO)	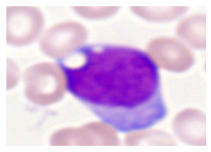
Lymphoblast-A (LYA)	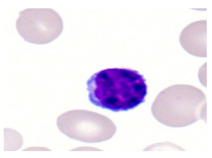	Promyeloblast (PMB)	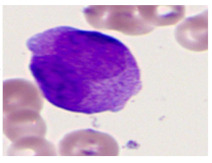
Erythroblast (EBO)	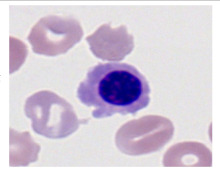	Neutrophil band (NGB)	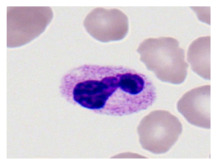
Keratinocyte stem cell (KSC)	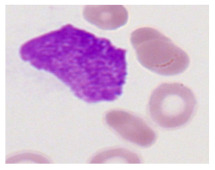	Neutrophil segmenter (NGS)	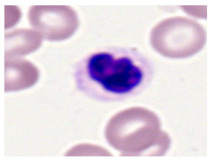
Myeloma cell Z (MMZ)	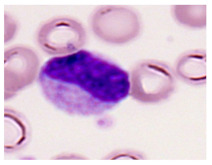	Promonocyte (PMO)	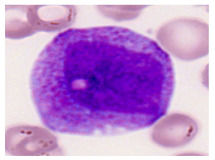
Basophil (BAS)	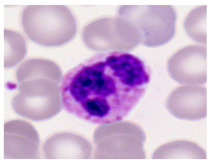		

**Table 2:** Performance of the proposed ResViT model for leukemia cell type on dataset-1.

Fold	Precision (%)	Recall (%)	F1-Score (%)	Kappa (%)	Misclassification	Accuracy (%)
Fold5	85.93	82.55	84.20	0.995	0.0033	99.67
Fold4	76.76	79.22	77.97	0.991	0.0065	99.35
Fold3	72.32	78.23	75.16	0.991	0.0063	99.34
Fold2	44.09	50.78	47.20	0.969	0.0212	97.87
Fold1	44.57	44.77	44.67	0.902	0.0681	93.19
Average	64.73	67.11	65.84	0.902	0.0086	97.88

**Table 3:** Performance parameters of the ResViT model on dataset-2 ALL.

Class	Recall (%)	Precision (%)	F1-Score (%)	OA-Kappa (%)	OA-Accuracy (%)
ALL-B	99.50	99.70	99.50	99.92	99.42
ALL-E	99.40	99.50	99.45		
ALL-PRE	99.49	99.00	99.24		
ALL-PRO	99.30	99.50	99.40		

**Table 4:** Summary of the performance of the proposed ResViT model with other existing models.

Author	Model	(No. of Images)	Accuracy (%)
Loey et al. [26]	AlexNet	2820	99.9
Pałczyński et al. [27]	MobileNet v2	260	97.4
Maaliw et al. [28]	InceptionResNetV2	100 (ALL-IDB1) 240 (ALL-IDB2)	99.60
Das et al. [29]	Orthogonal softmax layer (OSL) integrated with ResNet18	368	99.00
Wibowo et al. [30]	EfficientNetV2M CNN architecture and Bayes optimization	15,175	91.37
Lee et al. [31]	ResNet-50	241 patients	91.10
Verma et al. [32]	CNN-SVM hybrid	Private database	99.00
Das et al. [33]	DCNN (Inception-V3+FC)	15,135	97.34
Leng et al. [34]	Improved DETR	10,323	96.10 (precision)
Proposed	ResViT	Dataset-1 −18,236 Dataset-2 −20,000	99.67 99.42

**Table 5:** Class-wise performance of ResViT and the method by Matek et al. [35].

Class	Precision (Proposed vs. Matek et al.)	Recall (Proposed vs. Matek et al.)	F1-Score (Proposed Model)	No. of Images
BAS	100/48	96.64/82	100	79
EBO	100/75	60/87	100	78
EOS	100/95	100/95	100	424
KSC	100/53	100/77	50.00	15
LYA	50/20	100/7	66.67	11
LYT	100/96	96.97/95	98.46	3937
MMZ	100/90	49.33/90	49.14	1789
MOB	100/94	79.89/94	78.98	3268
MON	99.70/46	97.39/43	98.50	42
MYB	100/52	99.04/58	99.51	26
MYO	99.23/7	100/13	99.61	15
NGB	20/25	20/59	69.23	109
NGS	100/99	100/96	100	8484
PMB	40/45	100/54	57.14	18
PMO	100/63	75/54	85.71	70

**Table 6:** Performance comparison with the transformer-based methods.

Methods	Precision (%)	Recall (%)	F1-Score (%)	Kappa (%)	Accuracy (%)
Vaswani et al. [36]	61.19	60.28	60.74	85.94	90.40
Liu et al. [37]	62.72	63.51	63.11	88.73	93.48
Proposed	64.73	67.11	65.84	90.20	97.88

**Table 7:** Performance comparison with transformer-based methods on Dataset-2.

Methods	Precision (%)	Recall (%)	F1-Score (%)	Kappa (%)	Accuracy (%)
Vaswani et al. [35]	94.58	95.60	95.08	95.87	96.03
Liu et al. [36]	97.12	96.98	97.05	97.85	98.26
Proposed	99.41	99.92	99.40	99.92	99.43

**Table 8:** Effects of different components on model performance.

Dataset	Components	F1-Score (%)	Kappa (%)	Precision (%)
Dataset-1	ResNet-50	57.34	81.20	58.15
	ViT	60.18	85.47	61.08
	ResNet-50 + ViT	61.46	86.78	62.82
	ResNet-50 + dual stream ViT	65.84	90.20	64.73
Dataset-2	ResNet-50	92.18	93.07	93.58
	ViT	94.95	96.07	95.42
	ResNet-50 + ViT	96.29	97.08	97.83
	ResNet-50 + dual stream ViT	99.40	99.92	99.41

## Data Availability

The dataset used in the study can be downloaded from https://www.cancerimagingarchive.net/collection/aml-cytomorphology_lmu/ (accessed on 30 July 2024) and https://www.kaggle.com/datasets/mehradaria/leukemia/data (accessed on 10 August 2024).
